# *Aedes aegypti* feeding behavior during dengue outbreaks in two rural areas of Peru during the Yaku cyclone and El Niño phenomenon of 2023

**DOI:** 10.17843/rpmesp.2024.413.13930

**Published:** 2024-08-29

**Authors:** Archi Alejandro Ruiz-Polo, Rosa Elena Santillan-Valdivia, Cindy Yuriko Saavedra-Rios, Carlos Martin Nuñez-Rodriguez, Lya Emilia Niño-Mendoza

**Affiliations:** 1 Entomology Research and Training Center - CICE, Sub Regional Health Directorate Luciano Castillo Colonna, Sullana, Piura, Peru. Entomology Research and Training Center - CICE Sub Regional Health Directorate Luciano Castillo Colonna Sullana Piura Perú; 2 Faculty of Biological Sciences, Universidad Nacional Pedro Ruiz Gallo, Chiclayo, Lambayeque, Peru. Universidad Nacional Pedro Gallo Faculty of Biological Sciences Universidad Nacional Pedro Ruiz Gallo Chiclayo Lambayeque Peru; 3 Surveillance and Vector Control Program, Sub Regional Health Directorate Luciano Castillo Colonna, Sullana, Piura, Peru. Surveillance and Vector Control Program Sub Regional Health Directorate Luciano Castillo Colonna Sullana Piura Peru

**Keywords:** Aedes aegypti, Polymerase Chain Reaction, Polymorphism, Restriction Fragment Length, DNA

## Abstract

**Objective.:**

To determine the feeding behavior of Aedes aegypti in dengue outbreaks in two rural areas of Peru during the Yaku cyclone and El Niño phenomenon of 2023.

**Material and methods.:**

Eight blood samples (8 pools) were obtained from the abdomen of 80 Aedes aegypti specimens captured in the rural districts of Querecotillo and Marcavelica during the Yaku cyclone and El Niño dengue outbreaks. DNA was extracted from the analyzed samples, then a PCR was directed at the CytB gene as a genetic marker and the PCR products were enzymatically digested with the restrictases Hae III and Mwo I. The PCR-RFLP products were visualized by agarose gel electrophoresis at 4%.

**Results.:**

DNA was obtained from all samples and a 358 bp amplicon was obtained as a PCR product. Likewise, the only RFLP found in Hae III was from Homo sapiens sapiens (233 and 125 bp). RFLP was not found in Hae III of Gallus gallus and RFLP in Mwo I of Canis familiaris and Mus musculus.

**Conclusion.:**

Aedes aegypti showed conserved anthropophilic feeding behavior in dengue outbreaks in rural areas during the Yaku cyclone and El Niño.

## INTRODUCTION

*A. aegypti* usually feeds on humans (anthropophilia), a behavior demonstrated by studies on mosquitoes raised under laboratory conditions and mosquitoes captured in the wild ^1^^,^^2^. Only female *A. aegypti* mosquitoes can become infected with pathogens and transmit them to the host since they require blood to lay their eggs ^3^.

Having an anthropophilic behavior, *A. aegypti* does not depend on other vertebrates as food sources, therefore it is very predominant in urban areas since it does not need to feed on glycosidic compounds of plants ^4^^,^^5^. Because of this, it is the most efficient mosquito species for transmitting dengue virus ^6^.

On the other hand, there are also other species that feed on a wide range of vertebrates ranging from mammals to amphibians ^7^. However, recent studies indicate that populations of *A. aegypti* are adopting this behavior in rural areas, since specimens have been found feeding on humans, mongooses, birds, cattle, pigs, cats, rats and chickens ^8^.

It has long been demonstrated that *A. aegypti* has the capacity to colonize rural areas that act as breeding sites due to the large accumulation of containers, tires and other unusable items that provide ideal conditions for the development of the insect ^9^, complicating the formulation and adaptation of new strategies for the control of dengue, given that the dispersion and behavior of the vector has been studied very little in these areas ^10^, even more so during epidemic outbreaks of dengue framed in climatic phenomena in which there is a relatively high abundance of the mosquito population.

In this context, there are several extrinsic and intrinsic factors in the transmission of arboviruses to humans. Host location, temperature, humidity and food preference are extrinsic factors; and the circadian cycle, immune and tissue barriers that prevent virus replication and dissemination are the intrinsic factors ^11^^,^^12^.

It should be noted that studying the feeding behavior of *A. aegypti* is not only relevant because of the potential new reservoirs of arboviruses that could be found, but also because of significant physiological changes that could be acquired, such as the increase in the number of eggs per oviposition that has been demonstrated in specimens fed with *R. rattus* (rat) and *O. cuniculus* (rabbit), in comparison with *H. sapiens sapiens* (human) ^13^. The implementation of molecular methods to understand the feeding behavior of mosquitoes of public health interest is essential for the development of effective vector control strategies. This is the only way to understand the ecological dynamics of arbovirus transmission ^14^.

To date, there are no studies on the feeding behavior of *A. aegypti* during dengue epidemics in rural Peru during climatic events. Therefore, we aimed to determine the feeding behavior of *A. aegypti* during dengue outbreaks in two rural areas of Peru during the Yaku cyclone and in the El Niño phenomenon of 2023.

**KEY MESSAGES**

Motivation for the study. Dengue epidemics caused by *A. aegypti* occur during climatic events in tropical countries such as Peru; however, the feeding behavior of the mosquito usually goes unnoticed.

bold>Main findings*. A. aegypti* populations in Marcavelica and Querecotillo showed anthropophilic feeding behavior during cyclone Yaku and in the 2023 El Niño. However, populations with different feeding patterns are not ruled out.

Implications. The PCR-RFLP technique of the blood cell cytochrome B gene could be implemented in vector control policies through an entomo-virological surveillance plan.

## MATERIALS AND METHODS

### Study design

**Figure 1:**
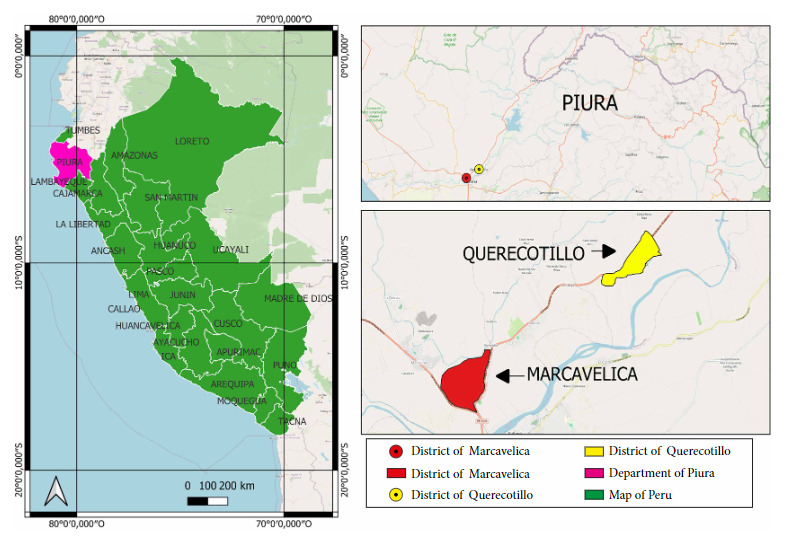
Geographical location of the districts of Querecotillo and Marcavelica where the *A. aegypti* specimens were captured.

This is a descriptive *in vitro* study conducted at the Molecular Biotechnology Laboratory of the Center for Research and Training in Entomology (CICE) of the Luciano Castillo Colonna Subregional Health Directorate, located in the district of Querecotillo, province of Piura, department of Piura, Peru. The study aimed to determine the feeding behavior of *A. aegypti* from blood samples collected from the abdomen of 80 specimens captured during the Yaku cyclone ^15^ and the 2023 El Niño ^16^, inside homes in the rural districts of Querecotillo (4°50′24″S/80°38′57″W) and Marcavelica (4°52′54″S/80°42′12″W) located in Sullana, Piura, Peru (Figure 1).

### Analyzed samples

The analyzed samples correspond to eight blood pools from the collections of the molecular biotechnology laboratory of CICE. Of the total number of samples, four belong to the district of Querecotillo (M1, M2, M3 and M4); and four to the district of Marcavelica (M5, M6, M7, M8). In Querecotillo, entomological captures were carried out on March 18; 19; 20; 21; 22 and 23, 2023; and in Marcavelica on June 2; 3; 4; 6 and 8, 2023.

### Entomological captures, specimen handling and blood collection

Mosquitoes and blood samples were collected by qualified personnel under strict entomological and conservation protocols for biotechnological applications. Initially, the personnel entered homes in Querecotillo and Marcavelica, then resting *A. aegypti* specimens were captured using entomological aspirators according to World Health Organization methodology ^17^. Immediately afterwards, the specimens were transferred to the bioassay area of CICE, where they were exposed to ethyl acetate impregnated with cotton for eight minutes, and taxonomically identified according to the Pan American Health Organization ^18^.

Specimen handling and blood collection was performed by selecting female mosquitoes that showed visible blood on the abdomen and discarding those that did not. The selected females were placed on sterile slides with 100 uL of DNA/RNA Shield Zymo Biomics preservative solution (R1100-250). Then, following an internal laboratory protocol, pressure was exerted with sterile toothpicks on the mosquito abdomen, the blood was expelled, mixed with the preservative solution, then the mixture was aspirated, and transferred to sterile microtubes with 200 uL of the same solution. Finally, the microtubes with the blood samples were stored at -20 ºC in the molecular biotechnology laboratory of the CICE.

### DNA extraction

DNA was extracted with the commercial Zymo BIOMICS DNA Miniprep Kit (D4300), the cell lysis step was modified with silica microbeads by a maceration with sterile plastic pistils, and 10,000 RPM centrifugation. Finally, we followed the manufacturer’s instructions.

### PCR of the CytB gene

PCR targeting the CytB gene was performed following the protocol proposed by Chena *et al*. ^14^, using the PCR kit GoTaq™ G2 Flexi DNA Polymerase (Promega M7801), and the primers proposed by Oshagi *et al*. ^19^ (Cytb 1: 5-CCCCTCAGAATGATATATTTGTCCTCA-3 and Cytb 2: 5́-CCATCATCCAACATCTCACTCAGCATGATGAAA-3). The reaction had a final volume of 50 µL, containing 22.5µL of nuclease-free water, 10 µL of buffer (1X), 3 µL of MgCL (1.5 mM), 1 µL of dNTPs (200 µM), 2.5 µL of Forward cyt b1 (10 uM), 2.5 µL of Reverse cyt b2 (10 uM), 0.5 µL of Gotaq Polymerase enzyme (1 U/reaction) and 8 µL of DNA. Thermal conditions and cycling consisted of an initial denaturation of 95 °C for 5 minutes, followed by 35 cycles with 95 °C for 30 seconds for denaturation, 58 °C for 30 seconds for hybridization, 72 °C for 1 minute for extension, a post-extension of 72 °C for 5 minutes and a storage temperature of 4 °C for up to 24 hours.

### Agarose gel electrophoresis

A 4% agarose gel electrophoresis was performed to validate the amplification of the CytB gene by PCR. Then, 3.6 grams of agarose was dissolved in 90 mL of 1X TAE buffer (Tris-Acetate-EDTA) and 4.5 uL of ethidium bromide (Thermo Scientific). A mixture of 4 uL of loading dye (6X DNA loading dye) and 10 uL of sample (PCR product) was loaded into each well of the gel. The electrical conditions for electrophoresis were 80 volts and 200 amps for 30 minutes.

### Enzymatic digestion of the CytB gene

Enzymatic digestion of PCR products was performed with H*ae* III and M*wo* I restrictionase enzymes recognizing polymorphic length restriction fragments (RFLP) of the CytB gene in H*ae* III from *H. sapiens sapiens* and *G. gallus*; and RFLP in M*wo* I from *M. musculus* and *C. familiaris* (Table 1).

**Table 1:** Restriction Fragment Length Polymorphism of the Cytb gene by restriction enzyme.

Vertebrate	RFLP	Restriction enzyme
*Homo sapiens sapiens* (human)	233/125	Hae III
*Gallus gallus* (chicken)	159/125/75	Hae III
*Canis familiaris* (dog)	187/114/57	Mwo I
*Mus musculus* (rodent)	187/171	Mwo I

RFLP: Restriction Fragment Length Polymorphism.

For the enzymatic reactions, we chose to follow the protocol described by Chena *et al*. ^14^, adapting the enzyme concentration prescribed in the H*ae* III and M*wo* I enzyme kits from New England Biolabs. We mixed 45 µL of PCR product, 20 µL of Buffer (1X) and 20 µL of restrictase enzyme (10 U/reaction) in the reaction of each enzymeThermal conditions for H*ae* III were 37 °C for 15 min for enzyme activation and 80 °C for 20 min for inactivation. Thermal conditions for M*wo* I were 60 ºC for 15 minutes for enzyme activity. Finally, 35 uL volumes of the enzymatic digestion product were visualized by 4% agarose gel electrophoresis.

## RESULTS

**Figure 2:**
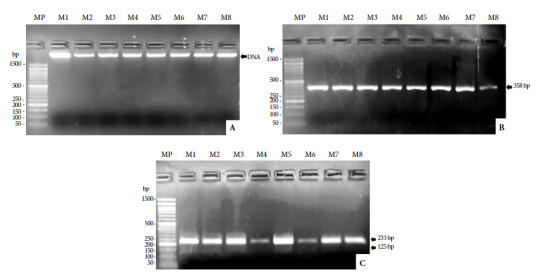
4% agarose gel electrophoresis. A. DNA extracted from blood cells from the abdomen of *A. aegypti*. B. PCR products of the CytB gene.C. RFLP of the CytB gene. MP: 100 bp molecular weight marker. M1: sample 1. M2: sample 2. M3: sample 3. M4: sample 4. M5: sample 5. M6: sample 6. M7: sample 7.

Figure 2A shows the DNA (fluorescent band) extracted from the blood cells of all the analyzed samples. Figure 2B shows the amplicons of the DNA extracted from all samples (358 bp). In Figure 2C, RFLP can be found in H*ae* III with molecular weights 233 and 125 bp corresponding to *H. sapiens sapiens*. No RFLP was observed in H*ae* III from *G. gallus* and no RFLP was observed in M*wo*I from *M. musculus* and *C. familiaris*.

## DISCUSSION

The feeding behavior of *A. aegypti* in dengue outbreaks during climatic phenomena such as the Yaku cyclone and El Niño has not been researched, in spite of its importance influencing the development of the vector and the transmission of the virus. It is important to highlight that the Yaku cyclone emerged and dissipated during the month of March 2023 ^16^, followed by El Niño ^15^ in June of the same year. Likewise, during these scenarios, dengue epidemics occurred in March ^20^^)^ and June ^21^. Therefore, since there was a difference of two months between March and June (April and May), the impact of the dengue epidemics could have been mitigated, given that the approximate life span of *A. aegypti* is 4 to 6 weeks ^22^. In addition, its eggs are highly resistant to desiccation and low temperatures for up to one year ^23^.

Total DNA extraction was achieved in all analyzed samples. mDNA was extracted from the white cells of all blood samples. White cells are nucleated and have organelles such as mitochondria ^24^. White blood cells are targets for molecular biotechnology applications in the medical field ^25^. The PCR products that we obtained were 358 bp amplicons, which is in agreement with the reports by Oshagi *et al*. ^19^ and Chena *et al*. ^14^.

Regarding food sources, RFLPs were found in H*ae* III from *H. sapiens sapiens* (Figure 2C); which determined that *A. aegypti* maintained an anthropophilic diet during the epidemic scenarios and climatic events in which they were captured. These findings are consistent with previous literature describing *A. aegypti* as a hematophagous and anthropophilic mosquito ^26^. According to the Pan American Health Organization ^27^, *A. aegypti* is characterized as a species with a strict diet of humans. Recent laboratory studies have reported that, due to the unavailability of humans as a food source, *A. aegypti* has fed directly on other vertebrates such as rats and rabbits ^14^, a behavior possibly caused by the availability of the anthropogenic food source. Other studies indicate that *A. aegypti* is a mosquito that also has a blood-feeding acceptance for vertebrates such as pigs, dogs, or chickens ^28^. In Thailand, *A. aegypti* populations have been found feeding on cattle, pigs, cats, rats, and chickens ^6^. Recent scientific evidence indicates that *A. aegypti* also feeds on domestic dogs, since dogs in rural and urban areas have been found infected with dengue virus (serotypes DENV2 and DENV3) ^29^. In Puerto Rico, two rural localities with *A. aegypti* populations fed on dog, cat, horse, and chicken blood have been reported ^30^.

The feeding preference of *A. aegypti* for humans is the result of an evolutionary process, since they were originally from African forests where they fed on the blood of wild animals and developed immature stages in trees ^31^^,^^32^. The anthropophilic habit of *A. aegypti* is due to its diurnal hematophagic activity and interaction with the host environment ^33^. The mosquito presents temporary genetic changes at the microgeographic level during dengue epidemic events, giving rise to genetic variability and eventual formation of subpopulations and subspecies with biological changes such as anthropophilic levels, biting behavior, insecticide resistance, vectorial capacity and competence ^34^^,^^35^. Feeding on vertebrates other than humans is influenced by hormone levels that vary throughout the life of the female mosquito ^36^.

This study has certain limitations. First, the number of analyzed vertebrates does not allow us to rule out the possibility that *A. aegypti* may have shown changes in feeding behavior during the studied climatic phenomena, since the methodology of the study does not describe RFLP for other species. Then, acrylamide gels were not used for the clear visualization of RFLPs with low molecular weight, being replaced by very concentrated agarose gels, causing that the RFLPs with higher molecular weight agglutinate and are more visible than those with low weight during electrophoresis. However, our study achieved its objective, being the first studying the feeding behavior of *A. aegypti* using molecular markers of blood cells during climatic phenomena in Peru.

In conclusion, *A. aegypti* maintained a conserved anthropophilic feeding behavior in Querecotillo and Marcavelica during the dengue outbreaks that occurred during the Yaku cyclone and during El Niño 2023, having found that humans were its only food source. However, feeding changes at some point in time and in another part of the country are not ruled out. Complementary research should focus on detecting dengue virus in all component phases of the biological cycle, and in domestic animals during climatic events, in order to determine vertical infections and potential reservoirs in massive populations of the mosquito. This study contributes to the validation of intradomiciliary strategies proposed and currently implemented for vector control in the analyzed rural areas, since changes in the feeding pattern of *A. aegypti* imply a variation in its intradomiciliary behavior.
