# Supplementary material for: Aedes aegypti feeding behavior during dengue outbreaks in two rural areas of Peru during the Yaku cyclone and El Niño phenomenon of 2023
Source: Rev Peru Med Exp Salud Publica. 2024 Aug 29;41(3):266–72. doi: 10.17843/rpmesp.2024.413.13930 (PMC11495925; doi:10.17843/rpmesp.2024.413.13930)
Supplement: Translation

# Translation: Comportamiento alimentario de *Aedes aegypti* en brotes de dengue de dos zonas rurales del Perú durante el ciclón Yaku y El Niño Global del 2023

**Authors:** Archi Alejandro Ruiz-Polo, Rosa Elena Santillan-Valdivia, Cindy Yuriko Saavedra-Rios, Carlos Martin Nuñez-Rodriguez, Lya Emilia Niño-Mendoza

**Affiliations:** 1 Centro de Investigación y Capacitación en Entomología - CICE, Dirección Sub Regional de Salud Luciano Castillo Colonna, Sullana, Piura, Perú.; 2 Facultad de Ciencias Biológicas, Universidad Nacional Pedro Ruiz Gallo, Chiclayo, Lambayeque, Perú.; 3 Programa de Vigilancia y Control Vectorial, Dirección Sub Regional de Salud Luciano Castillo Colonna, Sullana, Piura, Perú.

**Keywords:** Aedes aegypti, Reacción en Cadena de la Polimerasa, Polimorfismo de Longitud del Fragmento de Restricción, ADN

## Abstract

**Objetivos.:**

Determinar la alimentación del *Aedes aegypti* en brotes de dengue de dos zonas rurales del Perú durante el ciclón Yaku y El Niño Global del 2023.

**Material y métodos.:**

Se analizaron ocho muestras de sangre (8 pooles) obtenidas del abdomen de 80 especímenes *Aedes aegypti* capturados en los distritos rurales de Querecotillo y Marcavelica durante brotes de dengue acontecidos en el ciclón Yaku y en El Niño Global. Se extrajo ADN de las muestras analizadas, se llevó a cabo una PCR dirigida al gen CytB como marcador genético y los productos PCR fueron digeridos enzimáticamente con las restrictasas H*ae* III y M*wo* I. Los productos PCR-RFLP fueron visualizados por electroforesis en gel de agarosa al 4%.

**Resultados.:**

Se obtuvo ADN de todas las muestras y como producto PCR un amplicón de 358 pb. Así mismo, el único RFLP en H*ae* III observado fue el de *Homo sapiens sapiens* (233 y 125 pb). No se observó RFLP en H*ae* III de *Gallus gallus* y RFLP en M*wo* I de *Canis familiaris* y *Mus musculus.*

**Conclusión.:**

En brotes de dengue de zonas rurales, durante el ciclón Yaku y en El Niño Global, el *Aedes aegypti* presentó un comportamiento alimenticio antropofílico conservado.

## INTRODUCCIÓN

La preferencia alimenticia de *A. aegypti* se encuentra dirigida al ser humano (antropofilia), un comportamiento demostrado en estudios con mosquitos criados en condiciones de laboratorio y mosquitos capturados en su estado silvestre ^1^^,^^2^. Solo las hembras del mosquito *A. aegypti* pueden infectarse con patógenos y transmitirlos al huésped ya que requieren de sangre para poner sus huevos ^3^.

Al tener un comportamiento antropofílico, el *A. aegypti* no depende de otros vertebrados como fuentes de alimentación, dando lugar a su alta predominancia en áreas urbanas ya que no necesita alimentarse de compuestos glucosídicos de organismos vegetales ^4^^,^^5^. Debido a esto, es la especie de mosquito más eficiente para transmitir el virus del dengue ^6^.

Por otro lado, también hay otras especies que se alimentan de una amplia gama de vertebrados que van desde mamíferos hasta anfibios ^7^. Sin embargo, estudios recientes señalan que poblaciones de *A. aegypti* estarían adoptando dicho comportamiento en zonas rurales, ya que se ha encontrado especímenes alimentados de humanos, mangostas, pájaros, bovinos, cerdos, gatos, ratas y pollos ^8^.

Desde hace tiempo se ha demostrado que el *A. aegypti* tiene la capacidad de colonizar áreas rurales que actúan como focos de reproducción por la gran acumulación de contenedores, neumáticos y otros inservibles que brindan condiciones ideales para el desarrollo del insecto ^9^, generando una dificultad en la formulación y adaptación de nuevas estrategias para el control del dengue, dado que la dispersión y comportamiento del vector se ha estudiado muy poco en estas áreas ^10^, más aún durante brotes epidémicos de dengue enmarcados en fenómenos climáticos en los que existe una abundancia relativamente alta en la población del mosquito.

En ese contexto, existen diversos factores extrínsecos e intrínsecos en la transmisión de arbovirus a los humanos. La ubicación de los huéspedes, la temperatura, la humedad y la preferencia alimenticia serian factores extrínsecos; y el ciclo circadiano, las barreras inmunes y tisulares que impiden la replicación y diseminación del virus serian factores intrínsecos ^11^^,^^12^.

Cabe señalar que el estudio del comportamiento alimenticio del *A. aegypti* no sólo es relevante por los potenciales nuevos reservorios de arbovirus que podían encontrarse, sino también por cambios fisiológicos significativos que podrían adquirir, tal es el caso del aumento en el número de huevos por oviposición que se ha evidenciado en especímenes alimentados con *R. rattus* (rata) y *O. cuniculus* (conejo), en comparación con *H. sapiens sapiens* (humano) ^13^. Poner en práctica métodos moleculares que permitan comprender el comportamiento alimenticio de mosquitos con interés en salud pública es esencial para el planteamiento de estratégias efectivas en el control vectorial. Solo así se podrá entender la dinámica ecológica que existe en la transmisión de arbovirus ^14^.

Hasta la actualidad, no existen estudios sobre el comportamiento alimenticio de *A. aegypti* en epidemias de dengue en zonas rurales de Perú durante fenómenos climáticos. Por consiguiente, se planteó el objetivo de determinar la alimentación de *A. aegypti* en brotes de dengue de dos zonas rurales del Perú durante el ciclón Yaku y en El Niño global del 2023.

**MENSAJES CLAVE**

Motivación para realizar el estudio. En países tropicales como Perú, durante fenómenos climáticos surgen epidemias de dengue causadas por *A. aegypti*; sin embargo, el comportamiento alimenticio del mosquito usualmente pasa desapercibido.

Principales hallazgos. Las poblaciones de *A. aegypti* de Marcavelica y Querecotillo presentaron un comportamiento alimenticio antropofílico durante el ciclón Yaku y en El Niño Global del 2023. Sin embargo, no se descartan poblaciones con patrones alimenticios distintos.

Implicancias. La técnica PCR-RFLP del gen citocromo B de células sanguíneas podría implementarse en las políticas de control vectorial mediante un plan de vigilancia entomovirológica.

## MATERIALES Y MÉTODOS

### Diseño del estudio

El presente estudio es de tipo descriptivo *in vitro* realizado en el Laboratorio de Biotecnología Molecular del Centro de Investigación y Capacitación en Entomología (CICE) de la Dirección Subregional de Salud Luciano Castillo Colonna, ubicado en el distrito de Querecotillo, provincia de Piura, departamento de Piura, en Perú. El estudio consistió en determinar la alimentación de *A. aegypti* a partir de muestras sanguíneas recolectadas del abdomen de 80 especímenes capturados durante el ciclón Yaku ^15^ y el Niño global del año 2023 ^16^, en el interior de viviendas de los distritos rurales de Querecotillo (4°50′24″S/80°38′57″O) y Marcavelica (4°52′54″S/80°42′12″O) ubicados en Sullana, Piura, Perú (Figura 1).

**Figura 1.** Ubicación geográfica de los distritos de Querecotillo y Marcavelica donde se capturó los especímenes *A. aegypti* de los que se colectó la sangre analizada en el presente estudio.

### Muestras analizadas

Las muestras analizadas corresponden a ocho *pooles* de sangre pertenecientes a colecciones del laboratorio de biotecnología molecular del CICE. Del total de muestras, cuatro pertenecen al distrito de Querecotillo (M1, M2, M3 y M4); y cuatro al distrito de Marcavelica (M5, M6, M7, M8). En Querecotillo las capturas entomológicas se realizaron los días 18; 19; 20; 21; 22 y 23 del mes de marzo del año 2023; mientras que en Marcavelica los días 2; 3; 4; 6 y 8 del mes de junio del mismo año.

### Capturas entomológicas, manipulación de especímenes y obtención de sangre

Los mosquitos y las muestras de sangre se han recolectado por personal calificado bajo estrictos protocolos entomológicos y de conservación para aplicaciones biotecnológicas. Inicialmente, se ingresó a viviendas de Querecotillo y Marcavelica, luego se capturaron especímenes de *A. aegypti* en estado de reposo mediante aspiradores entomológicos de acuerdo con la metodología de la Organización Mundial de la Salud ^17^. Inmediatamente después, los especímenes fueron trasladados al área de bioensayos del CICE, donde se expusieron a acetato de etilo impregnado en algodón durante ocho minutos, y se identificaron taxonómicamente mediante claves de la Organización Panamericana de la Salud ^18^.

La manipulación de especímenes y obtención de sangre se realizó seleccionando mosquitos hembras que presentaron sangre visible en el abdomen y descartando aquellas que no la presentaron. Las hembras seleccionadas se montaron en láminas portaobjetos estériles con 100 uL de solución conservante DNA/RNA Shield Zymo Biomics (R1100-250). Luego, siguiendo un protocolo interno de laboratorio, se ejerció presión con mondadientes estériles sobre el abdomen del mosquito, se expulso la sangre, se mezcló con la solución conservante, se aspiró la mezcla, y se trasladó a microtubos estériles con 200 uL de la misma solución. Finalmente, los microtubos con las muestras de sangre se conservaron a -20 ºC en el laboratorio de biotecnología molecular del CICE.

### Extracción de ADN

Se extrajo ADN con el Kit comercial Zymo BIOMICS DNA Miniprep (D4300), se modificó el paso de lisis celular con microperlas de sílice por un macerado con pistilos plásticos estériles, y una centrifugación de 10 000 RPM. Finalmente, se siguieron las indicaciones del fabricante.

### PCR del gen CytB

Se realizó una PCR dirigida al gen CytB siguiendo las indicaciones del protocolo propuesto por Chena *et al*. ^14^, usando el kit PCR GoTaq™ G2 Flexi DNA Polymerase (Promega M7801), y los cebadores propuestos por Oshagi *et al*. ^19^ (Cytb 1: 5-CCCCTCAGAATGATATTTGTCCTCA-3 y Cytb 2: 5́-CCATCCAACATCTCAGCATGATGAAA-3). La reacción se trabajó con un volumen final de 50 µL, conteniendo 22.5µL de agua libre de nucleasas, 10 µL de buffer (1X), 3 µL de MgCL (1.5 mM), 1 µL de dNTPs (200 µM), 2.5 µL de Forward cyt b1 (10 uM), 2.5 µL de Reverse cyt b2 (10 uM), 0.5 µL de enzima Gotaq Polimerasa (1 U/reacción) y 8 µL de ADN. Las condiciones térmicas y el ciclaje consistieron en una desnaturalización inicial de 95 °C por 5 minutos, seguido de 35 ciclos con 95 ºC por 30 segundos para la desnaturalización, 58 °C por 30 segundos para la hibridación, 72 °C por 1 minuto para la extensión, una posextensión de 72 °C por 5 minutos y una temperatura de conservación de 4 °C por hasta 24 horas.

### Electroforesis en gel de agarosa

Se realizó una electroforesis en gel de agarosa al 4% que validó la amplificación del gen CytB por PCR. Se disolvieron 3,6 gramos de agarosa en 90 mL de tampón TAE 1X (Tris-Acetato-EDTA) y 4,5 uL de bromuro de etidio (Thermo Scientific). Se cargó una mezcla de 4 uL del colorante de carga (6X DNA loading dye) y 10 uL de muestra (producto PCR) en cada pocillo del gel. Las condiciones eléctricas de electroforesis fueron 80 voltios y 200 amperios por 30 minutos.

### Digestión enzimática del gen CytB

Se realizó una digestión enzimática de los productos de PCR con las enzimas restrictasas H*ae* III y M*wo* I que reconoce fragmentos de restricción de longitud polimórfica (RFLP, por sus siglas en inglés) del gen CytB en H*ae* III de *H. sapiens sapiens* y *G. gallus*; y RFLP en M*wo* I de *M. musculus* y *C. familiaris* (Tabla 1).

**Tabla 1:** Polimorfismo de Longitud del Fragmento de Restricción del gen Cytb por enzima de restricción empleada.

Vertebrado	RFLP	Enzima de restricción
*Homo sapiens sapiens* (humano)	233/125	Hae III
*Gallus gallus* (gallina)	159/125/75	Hae III
*Canis familiaris* (perro)	187/114/57	Mwo I
*Mus musculus* (roedor)	187/171	Mwo I

RFLP: Polimorfismo de Longitud del Fragmento de Restricción

En las reacciones enzimáticas se optó por seguir el protocolo descrito por Chena *et al.*^14^, adaptando la concentración enzimática prescrita en los kits enzimáticos H*ae* III y M*wo* I de New England Biolabs. En la reacción de cada enzima, se mezclaron 45 µL de producto de PCR, 20 µL de Buffer (1X) y 20 µL de enzima restrictasa (10 U/reacción). Las condiciones térmicas en H*ae* III fueron 37 ºC por 15 minutos para la activación enzimática y 80 ºC por 20 minutos para la inactivación. Las condiciones térmicas en M*wo* I fueron 60 ºC por 15 minutos para la actividad enzimática. Finalmente, volúmenes de 35 uL del producto de digestión enzimática se visualizaron por electroforesis en gel de agarosa al 4%.

## RESULTADOS

En la figura 2A, se observa el ADN (banda fluorescente) extraído de las células sanguíneas de todas las muestras analizadas. En la figura 2B, se observan los amplicones del ADN extraído de todas las muestras (358 pb). En la figura 2C, se observa RFLP en H*ae* III con pesos moleculares 233 y 125 pb que corresponden a *H. sapiens sapiens.* No se observó RFLP en H*ae* III de *G. gallus* y ni RFLP en M*wo*I de *M. musculus* y *C. familiaris.*

**Figura 2:** Electroforesis en gel de agarosa al 4%. A. ADN extraído de células de la sangre del abdomen de *A. aegypti.* B. Productos PCR del gen CytB. C. RFLP del gen CytB. MP: marcador de peso molecular de 100 pb. M1: muestra 1. M2: muestra 2. M3: muestra 3. M4: muestra 4. M5: muestra 5. M6: muestra 6. M7: muestra 7. M8: muestra 8.

## DISCUSIÓN

El comportamiento alimenticio de *A. aegypti* en brotes de dengue durante fenómenos climáticos como el ciclón Yaku y el Niño Global no se ha investigado, pese a que esto sería muy relevante por su influencia en el desarrollo del vector y la transmisión del virus. Es de importancia resaltar que el ciclón Yaku surgió y se disipó durante el mes de marzo del año 2023 ^16^, posteriormente en el mes de junio del mismo año se presentó El Niño Global ^15^. Así mismo, durante estos escenarios surgieron epidemias de dengue en el mes de marzo ^20^ y junio ^21^. Por tanto, sabiendo que entre marzo y junio existió una diferencia de dos meses (abril y mayo), el impacto de las epidemias de dengue pudo haberse mitigado, dado que el periodo de vida aproximado de *A. aegypti* es de 4 a 6 semanas ^22^. Además, de que sus huevos poseen una alta resistencia a la desecación y a las bajas temperaturas hasta por un año ^23^.

Se logró extraer ADN total de todas las muestras analizadas. La extracción de ADNm se realizó a partir de las células blancas de todas las muestras de sangre. Las células blancas son nucleadas y cuentan con organelos como las mitocondrias ^24^. Los glóbulos blancos son dianas para aplicaciones de biotecnología molecular en el campo de la medicina ^25^. Los productos PCR obtenidos fueron amplicones de 358 pb, el cual concuerda con lo señalado por Oshagi *et al.*^19^ y los reportados por Chena *et al.*^14^.

En cuanto a las fuentes de alimentación, se observaron RFLP en H*ae* III que corresponden a *H. sapiens sapiens* (figura 2C); lo cual determinó que *A. aegypti* mantuvo una alimentación antropofílica durante los escenarios epidémicos y fenómenos climáticos en los que se capturaron. Estos hallazgos coinciden con literatura publicada en la que se describe a *A. aegypti* como un mosquito hematófago y antropofílico ^26^. De acuerdo con la Organización Panamericana de la Salud ^27^, *A. aegypti* se caracteriza por ser una especie con una dieta estricta de humanos. Estudios recientes de laboratorio han reportado que, debido a la falta de disponibilidad del humano como fuente alimenticia, *A. aegypti* se ha alimentado directamente de otros vertebrados como ratas y conejos ^14^, un comportamiento posiblemente causado por la disponibilidad de la fuente alimenticia antropogénica. Otros estudios señalan que *A. aegypti* es un mosquito que también tiene una aceptación alimenticia por sangre de vertebrados como cerdos, perros o pollos ^28^. En Tailandia, se han encontrado poblaciones de *A. aegypti* alimentadas de bovinos, cerdos, gatos, ratas y pollos ^6^. Evidencia científica reciente señala que *A. aegypti* también se alimenta de perros domésticos, dado que se ha encontrado perros de zonas rurales y urbanas infectados con el virus dengue (serotipos DENV2 y DENV3) ^29^. En Puerto Rico, se han reportado dos localidades rurales con poblaciones de *A. aegypti* alimentados con sangre de perro, gatos, caballos y gallinas ^30^.

La preferencia alimenticia de *A. aegypti* por humanos es producto de un proceso evolutivo, ya que ancestralmente fueron originarios de bosques africanos donde presentaban una alimentación basada en sangre de animales silvestres, y un desarrollo de estadios inmaduros en los huecos de árboles ^31^^,^^32^. El hábito antropofílico de *A. aegypti* se debe a su actividad hematofágica diurna e interacción con el medio del huésped ^33^. Durante eventos epidémicos de dengue, el mosquito presenta cambios genéticos temporales a nivel microgeográfico, dando lugar a una variabilidad genética y una eventual formación de subpoblaciones, y subespecies con cambios biológicos como los niveles de antropofilia, comportamiento de picadura, resistencia a insecticidas, capacidad y competencia vectorial ^34^^,^^35^. La alimentación con vertebrados distintos al humano se encuentra influenciada por niveles hormonales que varían a lo largo de la vida del mosquito hembra, especialmente después de la emergencia ^36^.

El presente estudio tiene ciertas limitaciones. Primero, el número de vertebrados analizados no permite descartar que *A. aegypti* haya manifestado cambios en su comportamiento alimenticio durante los fenómenos climáticos en que se estudiaron; puesto que en la metodología empleada no se describe RFLP para otras especies. Segundo, no se utilizaron geles de acrilamida para la visualización nítida de RFLP con bajo peso molecular, siendo reemplazados por geles de agarosa muy concentrados, causando que durantre la electroforesis, los RFLP de mayor peso molecular se aglutinen y sean más visibles que aquellos con bajo peso. Sin embargo, se cumplió con el objetivo planteado, siendo la primera investigación en la que se ha estudiado el comportamiento alimenticio de *A. aegypti* empleando marcadores moleculares de células sanguíneas durante fenómenos climáticos en el Perú.

En conclusión, en Querecotillo y Marcavelica, durante los brotes de dengue presentados con el ciclón Yaku y en El Niño Global del 2023, el *A. aegypti* mantuvo un comportamiento alimenticio antropofílico conservado al haberse encontrado como única fuente alimenticia al humano. Sin embargo, no se descartan cambios alimenticios en algún punto del tiempo y en otra parte del país. Se necesitan investigaciones complementarias en las que el virus del dengue se detecte en todas las fases componentes del ciclo biológico, y en animales domésticos durante fenómenos climáticos, con el fin de determinar infecciones verticales y potenciales reservorios en las poblaciones masivas del mosquito. Este estudio contribuye a la validación de estrategias intradomiciliarias planteadas y ejecutadas actualmente en el control vectorial de las zonas rurales analizadas, ya que cambios en el patrón alimenticio de *A. aegypti* implican una variación en su comportamiento intradomiciliario.
